# “Workplace Physical Activity Program” (WOPAP) study protocol: a four-arm randomized controlled trial on preventing burnout and promoting vigor

**DOI:** 10.1186/s12889-019-6598-3

**Published:** 2019-03-12

**Authors:** Clément Ginoux, Sandrine Isoard-Gautheur, Philippe Sarrazin

**Affiliations:** Univ. Grenoble-Alpes, SENS, F-38000 Grenoble, France

**Keywords:** Work-related well-being, Burnout, Vigor, Physical activity intervention, Recovery mechanisms, Randomized controlled trial

## Abstract

**Background:**

WOPAP is a theoretically-grounded workplace physical activity intervention that aims to reduce work-related burnout and to improve vigor at work and other work-related outcomes. Using a randomized controlled trial, we investigate whether a 10-week program including two Nordic walking sessions per week is effective in improving employee well-being at work, in comparison with another attractive leisure activity (Theatre condition) or a waiting list control condition. The design of the study makes it possible to test the effect on burnout and vigor of the instructor’s style during physical activity (i.e., traditional vs. need-supportive style). Finally, this study is also interested in several possible psychological (i.e., detachment, relaxation, mastery, control, relatedness, and positive affects experiences) and physiological (i.e., cardiorespiratory fitness) mechanisms through which the practice of physical activity in the intervention could influence burnout and vigor.

**Methods:**

Employees of the authors’ University (*N* = 140) will be recruited via email, leaflets, and posters. Participants will be randomized to one of the four arms of the trial: (1) Physical Activity Traditional Style, (2) Physical Activity Need-Supportive Style, (3) Theatre condition, and (4) Waiting List Control. The experimental phase will last 10 weeks, followed by a six-month follow-up. During the ten weeks of the intervention, all groups – except the waiting list control – will carry out two activity sessions per week. Primary outcomes are burnout and vigor, secondary outcomes are work motivation, job satisfaction, work performance and work ability. These variables will be assessed before and after the intervention, and at three and six months after the end of the intervention. Moreover, burnout, vigor, needs satisfaction at work and psychological mediators will be assessed weekly throughout the intervention period.

**Discussion:**

If effective, this study will provide evidence for the promotion of workplace physical activity interventions including a need-supportive climate to improve employee well-being. Results could be used to design new research protocols, but also to implement more efficient programs in the workplace.

**Trial registration:**

ISRCTN12725337. Registered 21 March 2018. Registered retrospectively.

## Background

Recent national surveys showed that burnout – defined as “the gradual depletion over time of individuals’ intrinsic energetic resources, including the expression of emotional exhaustion, physical fatigue, and cognitive weariness” [1] – affects almost one fifth of the European workforce, and leads to negative consequences for employees and companies [2, 3]. It is associated with serious health issues, including impaired cognitive function, anxiety and depressive symptoms, increased cardiovascular risk and lower work productivity [4–6]. By contrast, vigor – defined as “one’s feelings of possessing physical strength, emotional energy, and cognitive liveliness” [7] – is related to job satisfaction and higher performance [8]. Given the benefits associated with vigor and the negative effects of burnout, workplace interventions are necessary to promote the former and prevent the latter. As Physical Activity (PA) has been consistently correlated with decreased burnout and increased vitality (for reviews, see [9–11]), offering employees the opportunity to do PA could be an efficient strategy to improve well-being at work.

### Physical activity and burnout/vigor at work

Cross-sectional and longitudinal studies have shown a negative relationship between burnout and PA on one hand, and a positive relationship between vigor and PA in the other; employees regularly engaging in PA report lower burnout symptoms and higher vigor levels than physically inactive employees [12–15]. Also, diary studies indicated that PA can decrease work-related fatigue and increase vigor on a daily level [16–18]. Given these promising results, a handful of studies have examined the effectiveness of workplace PA interventions and revealed a significant decrease in burnout [19–21] among employees who engage in workplace PA interventions. Despite these first results, some shortcomings and blind spots in the empirical evidence prevent a complete understanding of the PA-burnout/vigor relationship. First, many studies are correlational in nature [12, 22] and causality cannot be reasonably determined. Second, the few available intervention studies suffer from several methodological concerns, such as the lack of a control group [20, 23], no randomization [20, 21], or no objective measure of PA intensity during sessions [21, 23, 24]. More important, some studies mixed PA and “less active” leisure activities or other interventional strategies in their programs (i.e. yoga or motivational interviewing sessions to facilitate daily PA and relaxation, cognitive-behavioral stress management programs) making it impossible to identify the real “PA” contribution in the observed effects [23, 25–27]. Is it the PA or doing another activity that causes a decrease in burnout? In the same vein, not all the intervention studies control for the effects of the instructor’s motivational style that supervises PA sessions [28]. Consequently, it is not possible to know if it is the PA, the instructor’s motivational style or both that are responsible for the effects observed. Finally, the mechanisms of the PA-burnout/vigor relationship remain partially unknown because all the interventions except two [28, 29] did not investigate mediators which could explain the effect of PA on work well-being.

In view of this background, the aim of the present study is threefold. Firstly, to test the effects of a workplace PA intervention on work-related burnout and vigor using a design that allows for strong causal inferences and rules out alternative explanations. Specifically, the objective is to examine if a 10-week PA program including two Nordic walking sessions per week could reduce burnout and improve vigor at work among employees with moderate-to-high levels of burnout who do not participate in sport regularly, in comparison with another attractive leisure activity – i.e., Theatre condition – or a waiting list control condition. The second objective is to capture the effect of the instructor’s motivational style when supervising PA, by testing if a psychological needs supportive motivational style increases the benefits of PA on burnout and vigor at work, compared to a traditional motivational style. The third objective is to investigate the mediators of the relation between PA and burnout/vigor at within- and between-subject levels, in order to improve knowledge on the mechanisms explaining the effect of PA on well-being at work.

### PA, expressive activities and burnout/vigor at work

Nordic walking was chosen because while it allows moderate to vigorous energy expenditure [30], it does not require particular physical qualities, or expensive equipment. Moreover, this activity has been identified as efficient in decreasing fatigue states [31] and there are numerous examples of the successful implementation of walking interventions in the work setting (for a review, see [32]). Comparing participants doing Nordic walking with those in the waiting list control condition enables us to test the effect of PA on burnout and vigor compared to the natural development of these affective states during the same period at work. Based on available research, we hypothesize:

- *Hypothesis 1a:* the PA intervention is effective in reducing burnout and/or improving vigor at work compared to the waiting list control condition.

In addition to the PA condition, we have included an expressive activity condition (Theatre condition) to compare the effects of two leisure activities, with only one requiring high energy expenditure (Nordic walking). Sonnentag and her colleagues recently recommended that future studies should examine whether employees who engage in creative and cultural activities in their free time report a higher level of well-being thereafter, and recover faster and more effectively from work, than other employees [33]. Indeed, some correlational studies [34, 35] showed positive relationships between the frequency of creative and cultural activities during leisure time and recovery experiences and work engagement. As far as we know, no workplace interventions have examined the effectiveness of expressive activities or compared effects of PA and expressive activities on burnout/vigor. While both activities induce psychological mechanisms explaining improvements in work-related well-being (see below), regular PA leads to additional biological adaptations which could contribute to reduced physiological reactions to stressors [36, 37]. The combination of these psychological and physiological benefits in PA could lead to higher effects on burnout/vigor compared to expressive activities. Consequently, we expect that:

- *Hypothesis 1b:* the Theatre condition intervention is effective in reducing burnout and/or improving vigor at work
- *Hypothesis 2:* PA is more effective than Theatre condition in reducing burnout and/or improving vigor at work

### Quality of PA experience: Role of the instructor’s need-supportive style

Several studies show that the experience quality associated with leisure activities, namely the extent to which these activities are perceived as positive and pleasurable, is important to consider in order to have a thorough understanding of the recovery potential of an off-job activity [38–40]. More precisely, level of recovery from work will be enhanced when employees engage in leisure activities that they enjoy and do in a self-determined way. In this respect, according to Self-Determination Theory (SDT; [41]) an instructor’s motivating style is central to the quality of an individual’s experience. When instructors support satisfaction of the three basic psychological needs for autonomy (the need to experience a sense of choice and freedom to engage in an activity), competence (the need to feel able to effectively carry out challenging tasks) and relatedness (the need to develop meaningful relationships with the social environment and acceptance by significant others), they substantially increase the chances that individuals will have a positive experience. By contrast, when instructors do not support, or even worse, frustrate individuals’ psychological needs, they maximize the chances that individuals will have a negative experience that will have no recovery effect. Intervention studies [42, 43] have shown that it is possible to train exercise instructors to use a need-supportive style in order to maximize exercisers’ self-determined motivation and well-being. To our knowledge, there is only one workplace PA intervention in which an exercise instructor has been trained to deliver an autonomy need-supportive style [28]. It has proven to be effective in improving work-related well-being, by reducing feelings of fatigue and improving participants’ subjective vitality. However, as underlined above, the study design does not make it possible to determine whether the observed effects are due to PA and/or to the instructor’s need-supportive style. In order to disentangle the PA effect from the instructor’s style effect, we will implement two PA conditions: one supervised by an instructor trained to deliver a “traditional” motivational style, and the other supervised by an instructor trained to deliver a need-supportive style. In light of previous literature, we hypothesize that:

- *Hypothesis 3:* the positive effect of PA on burnout and/or vigor will be more pronounced when instructors use a need-supportive style rather than a “traditional” motivational style.

### Physical Activity and Secondary Outcomes

In addition to burnout and vigor, we will assess the consequence of the different conditions on four secondary outcomes, to check whether PA might also have other positive consequences on important job-related variables. First, we will examine the effects of the intervention on *job satisfaction*. This variable refers to a “pleasurable or positive emotional state resulting from the appraisal of one’s job or job experiences” [44]. Thøgersen-Ntoumani et al. [28] showed that participants in a workplace PA program experienced greater levels of enthusiasm and relaxation at work during the afternoon when they carried out PA during lunchtimes compared to the days when they did not. Then, by referring to SDT [45, 46], we will examine the effect of the intervention on the quality of *work motivation*, namely on autonomous (when the professional activities are experienced as emanating from one’s self) or controlled (when they are experienced as emanating from internal or external pressures) motivation. Such motivations are important to study because they are related to a host of cognitive, affective and behavioral consequences such as well-being, job satisfaction, organizational commitment, workaholism, burnout and turnover intentions [47, 48]. We will also investigate the effect of PA intervention on *work ability*. This refers to the “physical, psychological, and social capability of a worker to perform and interact within their work, and the individual’s specific work demands, health conditions, and mental resources” [49]. It has been demonstrated that burned-out people report decreased work ability [50], and an intervention study [37] reported that a six-week physical activity program improved work ability. Finally, we will study the effect of the intervention on the self-reported *work performance*. This variable refers to work-related activities expected of an employee and how well these activities were executed [51]. A few previous studies [52, 53] have reported that more physically active employees reported higher self-rated work productivity, and that a walking program in the workplace over sixteen weeks [54] increased work-performance by the end of the intervention.

- *Hypothesis 4:* the PA intervention is effective in improving job satisfaction (H4a), autonomous work motivation (H4b), work ability (H4c), and work performance (H4d).

### Psychological and Physiological Mechanisms of the PA – Burnout/Vigor Relationship

Although beneficial effects of PA on work-related well-being have been demonstrated, mechanisms underlying these effects remain largely unexplored. Drawing from several theoretical frameworks, at least seven plausible hypotheses can be invoked to explain this relationship: psychological detachment, relaxation, mastery, control, relatedness, positive affects, and cardiorespiratory fitness. Sonnentag and Fritz [55] used the concept of “recovery experiences” to characterize attributes associated with off-job activities contributing to work recovery. They distinguish four experiences: psychological detachment, relaxation, mastery and control. According to the Effort-Recovery Model [56], *psychological detachment* (i.e., a subjective experience of leaving work behind, “switching off”, and forgetting about work during non-work time) and *relaxation* (i.e., a state of low activation that poses no demands on the psychobiological system) may be helpful in recovering because while they are being experienced they protect the employees from experiencing the same acute load responses (e.g., accelerated heart rate, elevated blood pressure levels, stress) as those they have during the workday. On the other hand, according to the Conservation Of Resources theory [57], the restoration of personal resources that are lost when engaging in work-related effort constitutes another important element to the recovery process [55]. In this respect, *mastery* (i.e., pursuing off-job activities that offer challenging tasks and the opportunity to learn new skills and to experience success) and *control* (i.e., the degree to which a person can decide which activity to pursue during leisure time, as well as when and how to pursue this activity; [55]) experiences may help recovery because they restore and/or build up depleted internal resources. It is striking to note the conceptual overlap between mastery and control experiences on one hand, and the competence and autonomy need satisfaction presumed by SDT [41], on the other hand. Since SDT proposes the existence of a third need – namely, *relatedness* – whose satisfaction is likely to facilitate individuals’ well-being and optimal functioning, some scholars [58] hypothesize that a sense of relatedness constitutes another positive experience likely to restore resources that have been depleted during work.

In addition to the five previous psychological recovery experiences, the *positive affects* that individuals derive from their off-job activities were shown to be related to daily recovery [38]. More precisely, according to the Broaden-and-Build theory [59] and the Conservation Of Resources theory [57], positive emotional states such as happiness or feeling energetic, experienced during off-job activities have the potential to stop the prolongation of negative states built up during the work day, and to build personal resources facilitating the work recovery process. Since meta-analytic data demonstrate that PA is consistently associated with increased energy [60] and positive affects [61], emotional spillover from participation in such activities could be a valuable short-term strategy to promote work recovery [62].

Finally, physiological mechanisms can also be mentioned to explain the beneficial effects of PA. According to the cross-stressor adaptation hypothesis [63], regular PA induces biological adaptations which reduce physiological reactions to all stressors, whether related to PA or more general. Cumulative evidence indicates that employees with higher *cardiorespiratory fitness* (i.e., the maximal aerobic power of an individual, reflected by the maximal oxygen uptake; [64]) regulate and cope more efficiently with their stress [65].

Based on this evidence, we formulate the following hypotheses:

- *Hypothesis 5:* The effects of the PA intervention on burnout and/or vigor at work are mediated by higher psychological detachment from work (H5a), relaxation (H5b), mastery (H5c), control (H5d), relatedness (H5e), positive affects (H5f) experiences, and cardiorespiratory fitness (H5g).

### Weekly trajectories of employee burnout, vigor, and psychological recovery mechanisms

Following the recommendations by de Vries and colleagues [29, 66], we will examine the employees’ well-being and recovery trajectories during the course of the trial in order to answer two questions: (a) what is the minimum exposure to exercise or Theatre condition required for burnout and vigor to differ between interventions and control conditions? and (b) are the feeling of having recovered and the experiences of recovery among participants in PA and Theatre condition related to differences in their vigor and burnout trajectories? To be able to answer these questions and provide a finer grain of analysis on the development of well-being indicators during the intervention and on the relationships between these indicators and the recovery mechanisms, during each week of the intervention we will assess the primary outcomes (burnout, vigor) and the psychological mechanisms of recovery (detachment, relaxation, mastery, control, relatedness, and positive affects experiences) using single-item scales. While it is difficult to predict precisely when the well-being indicators will change as a function of the intervention, we nevertheless expect that:

- *Hypothesis 6a:* Participants in the PA and Theatre conditions report a decrease in feelings of burnout and/or an increase in vigor each week over the intervention period compared to participants in the control condition.
- *Hypothesis 6b:* Participants in the PA-need-supportive style conditions report a decrease in feeling of burnout and/or an increase in vigor each week over the intervention period compared to participants in the PA-traditional style and Theatre conditions.

Regarding the relationships between recovery and burnout/vigor trajectories, we expect that:

- *Hypothesis 6c:* Participants in intervention groups who experience greater feelings of psychological detachment from work, relaxation, mastery, control, relatedness, or positive affects experiences during the sessions, show a larger improvement in their weekly burnout and/or vigor trajectories compared to participants who experience lower feelings during these sessions.

### Moderator effects of compliance

We will also investigate the effect of participants’ compliance to the program on burnout/vigor. Compliance refers to the frequency with which the participants attend the activity sessions during the length of the program. Studies [37] had shown that the most assiduous participants benefit more from the effects of the intervention and report higher work-related well-being at the end of the intervention, compared to less assiduous participants. Therefore, we hypothesize that:

- *Hypothesis 7:* The positive effect of PA on burnout and/or vigor will be more pronounced among participants with higher compliance to activity sessions compared to participants with lower compliance.

### Control variables

It has been widely demonstrated that the work environment and its characteristics can influence employee well-being. For instance, studies have shown that some psychological risk factors in the workplace positively or negatively predict burnout and vigor [67]. In the same vein, research grounded in SDT has shown that a work environment supporting the employees’ three basic psychological needs helps develop autonomous motivation and well-being at work while an environment that frustrates these needs is related to controlled motivation and employee ill-being (for a review, see [68]). As a result, we will control for the job characteristics and the employees’ work need satisfaction in order to test the true effect of the intervention on the changes in job well-being.

## Methods

### Study design

**Fig. 1:**
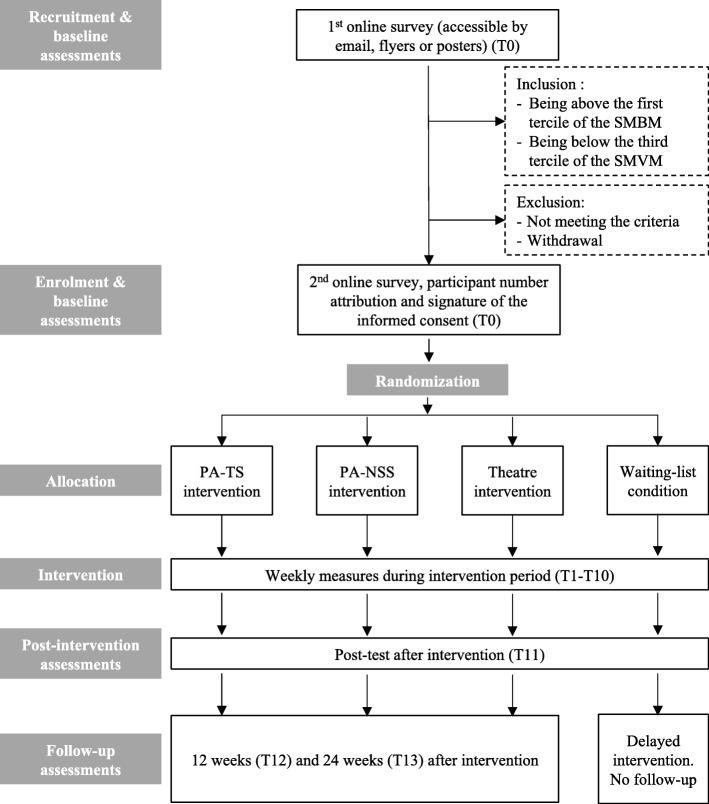
Protocol flowchart

This study will be a four-arm parallel randomized controlled trial: the ‘PA traditional style’ (PA-TS), the Theatre, the ‘PA need-supportive style’ (PA-NSS) and the Waiting List Control (WLC) conditions. See flowchart (Fig. 1) for an overview of the study design.

### Ethical issues

The research plan has been approved by the third French South Mediterranean Protection of Persons Ethics Committee (Registration number: 2017.03.02bis). Informed consent for participation in the study and a medical certificate authorizing walking-based PA (only for PA groups) will be obtained from participants.

### Recruitment process

Participants will be employees of the authors’ University. They will be recruited through email, posters and flyers posted on the university campus. Basic information about the intended program will be provided and those interested will be invited to complete a short online survey assessing their burnout and vigor at work, usual PA and some demographic information. Based on existing cut-off scores on the Shirom Melamed Burnout Measure (SMBM) and the Shirom Melamed Vigor Measure (SMVM) (obtainable at http://www.shirom.org/arie/index.html), inclusion criteria for participation will be: a) being above the first tercile of the SMBM (i.e., scoring ≥1.79 on the SMBM Burnout total score), b) being below the third tercile of the SMVM (i.e., scoring ≤ 5.00 on the SMVM Vigor total score). Exclusion criteria will be a) employees currently receiving or having received in the last six months pharmacological treatment for mental health disorders, b) having contraindications to exercise, and/or c) engaging in regular demanding physical training for competitive sports (i.e., Level 4 using the Saltin-Grimby PA Level Scale; see [69]). Eligible participants will receive a randomly assigned participant number and the informed consent form that they will have to read and sign. They will also be invited to complete a second online survey assessing some of the baseline measures: job characteristics, recovery experiences during leisure activities, work ability, job satisfaction, work performance. Participants will be randomized to one of the four conditions (see below). Then twice a week for ten weeks participants in the three activity conditions will take part in activities on the university campus. In the first session, a baseline cardiorespiratory fitness test will be conducted for all the participants.

### Sample size

Sample size calculations were based on an a priori power analysis based on a previous study carried out on similar primary outcomes in the workplace [37, 66]. This study has reported a positive effect of the PA intervention on the emotional exhaustion component of burnout, with a small effect size (η^2^ = 0.03; f = 0.176). According to this result, we conducted an analysis with G*Power software [70] examining the evolution of burnout and vigor depending on the conditions as inter-individual factors, with an moderate effect size of d = 0.35, a statistical power of 80%, a threshold of significance at *p* < 0.025 considering our two primary outcomes, a correlation of .5 across repeated measures, and a potential dropout rate of 25% for a 10-week program with 4 groups. The results of this analysis recommend recruiting a minimum of 140 participants, 35 per group.

### Randomization and blinding

Participants will be randomly assigned to one of the four conditions. A laboratory engineer will carry out the randomization once all participants are included, using the random function of Excel® software. Then, a second randomization will be done with the next 60 participants. A laboratory engineer will carry out the randomization, using the random function of Excel® software. In order to reduce bias, participants in PA conditions will not know their motivational style allocation (i.e. PA-TS or PA-NSS), and the instructor of each PA condition will not know the existence of the other condition. Finally, research assistants collecting data will be blinded to the treatment allocation.

### Conditions

#### PA traditional style condition

The PA-TS condition will be composed of twenty sessions of Nordic walking over ten weeks. Participants will walk twice a week in a group of twelve people. Each session will last 60 min, including welcome, warm-up, practice, cool-down and stretching. The instructor of the PA condition (a bachelor’s degree student in sports sciences) will have been trained to teach Nordic walking to beginners as this physical activity is taught in textbooks [71]. We called this condition ‘traditional’ because the instructor will only be trained to teach the correct walking technique and will not receive any training to support the basic psychological needs of participants. He/she will receive a program plan describing the warm-up, cool-down and stretching exercises, places and the duration of the Nordic walking itself for each session.

#### PA need-supportive style condition

In the PA-NSS condition, the organization and content of the session will be identical to the PA-TS condition. The main difference will be the motivational style implemented by the instructor. Two bachelor’s degree students in sport sciences will be both trained to deliver Nordic walking sessions using a need-supportive communication style. Grounded in SDT, the training will consist of four one-hour sessions based on SDT training [42, 72, 73] used in previous studies. In these workshops, instructors will be introduced to the main SDT postulates, will analyze video examples to identify strategies that are supportive to, and those which might frustrate, participants’ needs, and will create strategies to support participants’ needs in Nordic walking with the help of a research team member. For example, instructors will be trained to implement need-supportive strategies, such as: providing choice (e.g. type of exercise, intensity, place, route), encouraging participant input, feedback and questions; giving meaningful explanations; being empathetic and acknowledging difficulties, negative feelings or objections; being accessible, attentive and caring; trying to motivate by promoting enjoyment and the personal value of exercise or giving precise and positive feedback.

#### Theatre condition

The Theatre condition will be composed of twenty sessions of theatre classes over ten weeks (twice a week), in a group of 15 people guided by a master’s degree student in performing arts. Each session will last 60 min, and begin with warm-up voice exercises followed by oral, theatre or improvisation exercises. The theatre sessions will include public speaking games, exercises to use the body according to context and emotions, breathing and pronunciation exercises, confidence exercises in pairs, and an introduction to improvisation exercises.

#### Waiting list control condition

During the ten weeks of the three interventions, participants in the WLC condition will not carry out an activity. They will be contacted each week by email to complete an online questionnaire assessing measures similar to those for the three other groups. At the end of the period, they will be offered the possibility of starting the intervention in a delayed group the following year.

### Measures

Table 1 provides an overview of the measurement points of the primary and secondary outcomes, mediators, moderators and control variables, and manipulation check variables. At the end of the second session of each week, participants will complete a questionnaire measuring their weekly burnout, vigor, needs satisfaction at work and workload, as well as recovery mechanisms experienced, affects and effort perception during sessions (only for the 3 intervention groups). Five minutes are requested to complete the questionnaire. In week six of the program, participants will complete an online survey measuring job characteristics, and intervention groups will wear a 3-axis accelerometer to assess their energy expenditure during PA or theatre sessions. In week eight of the intervention, all instructors will be recorded audio-visually and participants will complete an online survey assessing their instructor’s motivational style during the sessions. At the beginning of the final session of the intervention participants will replicate the cardiorespiratory fitness test, and at the end of the intervention, participants will complete an online survey measuring the same variables as those assessed at baseline. Finally, three and six months after the end of the intervention, participants will be invited to complete the follow-up online questionnaire. In order to measure the *compliance* to the program, instructors from the three intervention groups will be asked to indicate the participants’ presence or absence at each session in a register.

**Table 1:** Overview of the measurement points for the primary and secondary outcomes, during intervention and weekly measures, and follow-up measurements

	Variables	Pre-intervention (T0)	During intervention (T6 or T8)	Weekly (T1-T10)	Post-intervention (T11)	Follow-up at 3 and 6 months (T12–13)
Primary outcomes	− Burnout	✓		✓	✓	✓^a^
	− Vigor	✓		✓	✓	✓^a^
Secondary outcomes	− Job satisfaction	✓			✓	✓^a^
	− Work motivation	✓			✓	✓^a^
	− Work performance	✓			✓	✓^a^
	− Work ability	✓			✓	✓^a^
Psychological and physiological mediators	− Off-job recovery activities	✓			✓	
	− Psychological detachment	✓		✓^a^	✓	
	− Relaxation	✓		✓^a^	✓	
	− Control	✓		✓^a^	✓	
	− Mastery	✓		✓^a^	✓	
	− Relatedness	✓		✓^a^	✓	
	− Positive Affects			✓^a^		
	− Cardiorespiratory Fitness	✓			✓	
Moderator variable	− Compliance to activity sessions			✓^a^		
Control variables	− Job characteristics	✓	✓(T6)	✓^b^	✓	
	− Needs satisfaction at work	✓	✓(T6)	✓	✓	
	− Workload at work			✓		
Manipulation check	− Instructors observed motivating style		✓(T8)			
	− *participants’ perception of the instructor’s motivating style*		✓(T8)			
	− Objective effort during sessions		✓(T6)			
	− Perceived effort during sessions			✓^a^		

^a^Variables only collected in intervention groups; ^b^Variables only collected in the waiting-list group

#### Primary outcomes

##### Burnout at work

*Burnout* will be assessed with the French version [74] of the SMBM [5]. The scale consists of 14 items divided into three subscales: *physical fatigue* (e.g., “I feel physically drained”), *cognitive weariness* (e.g., “I feel I’m not thinking clearly”), and *emotional exhaustion* (e.g., “I feel I am not capable of investing emotionally in coworkers and customers”). Scores are rated using a seven-point Likert scale (1 = never; 7 = always). In prior research, the English [22, 75, 76] and French [74] version of the SMBM has proved to be a valid and reliable instrument to assess burnout. Using data from the baseline measurements, we will select the three most representative items according to their loading on each of the three factors to assess weekly burnout.

##### Vigor at work

V*igor* will be measured with the French version [77] of the SMVM [7]. The scale consists of 12 items divided into three subscales: *physical strength* (e.g., “I feel I have physical strength”), *cognitive liveliness* (e.g., “I feel able to be creative”), and *emotional energy* (e.g., “I feel able to show warmth to others”). Scores are rated using a seven-point Likert scale (1 = never; 7 = always). In prior research, the English [78–80] and French [77] version of the SMVM has proved to be a valid and reliable instrument to assess vigor at work. Using data from the baseline measurements, we will select the three most representative items according to their loading on each of the three factors to assess weekly vigor.

#### Secondary outcomes

##### Job satisfaction

To measure employees’ job satisfaction the five items from the Global Professional Life Satisfaction scale [81] will be used. This scale measures overall job satisfaction as felt by the individual with a seven-point scale (1 = strongly disagree, 7 = strongly agree) (e.g., “I’m satisfied with my professional life”). Studies have reported good reliability and validity of this scale [81, 82].

##### Work motivation

To measure employees’ work motivation we will use the French version of the Motivation at Work Scale [83]. The scale consists of four subscales of three items: *intrinsic motivation* (e.g., “I do this job for the moments of pleasure that this job brings me”), *identified regulation* (e.g., “I do this job because this job fulfils my career plans”), *introjected regulation* (e.g., “I do this job because my reputation depends on it”) and *external regulation* (e.g., “I do this job for the paycheck”). Scores will be rated on a 7-point Likert scale (1 = totally disagree; 7 = totally agree). The reliability of this scale has been demonstrated by confirmatory factor analyses and evidence for validity was satisfactory [83].

##### Work performance

Global work performance will be assessed with an item from the World Health Organization Health and Work Performance Questionnaire (WHO HPQ; [51]), “How would you rate your overall performance on the days you worked during the past 7 days?” (0 = the worst anyone can do, 10 = the very best that top workers in a job like yours can do). A single item such as this has been used in similar studies [54].

##### Work ability

Work ability will be measured with a single item [49]: “Can you indicate how you rate your current work ability when you compare it with your lifetime best?” (0 = completely unable to work, 10 = work ability at its best). This item has been identified as a good alternative to more exhaustive measures of work ability [49] and has been used in a similar study [66].

#### Psychological and physiological mediators

##### Off-job recovery activities

To identify whether participants engage during their free time in activities with recovery potential - including those proposed in the study - we will ask them to answer the following question, “How often did you spend your time doing this off-job activity, in the last four weeks?” (1 = never, 7 = 6–7 times per week). We will focus on the four activities the most related to well-being and recovery [33, 84]: low-effort activities (e.g., watching TV, lying on the sofa), social activities (e.g., meeting others, making a phone call in order to chat), physical activities (e.g., keep-fit, cycling, dancing), and creative and expressive activities (e.g., theatre, playing music, singing). Then, following the procedure used by Korpela and Kinnunen [84], we will ask the participants, “How effective for recovery from work is the time spent on this off-job activity?” (1 = not at all effective, 7 = highly effective).

##### Psychological recovery experiences

To accurately identify the psychological experiences that may underlie the recovery process, we will use a French adapted version of the Recovery Experience Questionnaire (REQ; [55]). Participants will have to answer to the stem: “In the last 4 weeks, to what extent would you say that the activities you did during your free time (those identified above), enabled you […]”. The items comprise four dimensions: *psychological detachment* (4 items, e.g., “[…] to forget work”), *relaxation* (4 items, e.g., “[…] to kick back and relax”), *control* (4 items, e.g., “[…] to decide for myself what to do”), and *mastery* (4 items, e.g., “[…] to learn new things”). In order to assess the *experience of relatedness* [58], the REQ items will be completed with a French adapted version of the relatedness subscale of the Balanced Measure of Psychological Needs [85] (4 items, e.g., […] to feel close and connected with one or more people who participated with you in these activities). The answers will be provided on a Likert-type scale ranging from (1) *strongly disagree* to (7) *strongly agree*. Factorial validity and reliability will be tested in a pilot study. Using data from the baseline measurements, we will select the most representative items of each of these five dimensions according to their loading on each of the five factors to assess weekly psychological recovery experiences (see below).

##### Cardiorespiratory fitness

We will measure cardiorespiratory fitness using a forty-five second squat test [64]. After a five-minute rest period in a lying position, participants will have to stand up and stay in a standing position for a few seconds until their heart rate (HR) stabilizes. Then they will perform 30 squats in 45 s, following the tempo of 80 beats min^− 1^. The squatting movement will consist of 90° flexion of the knees, keeping the heels on the ground and the back straight, and the arms extended forwards. At the end of the squatting period, participants will lie down and recover for 3 min. The heart rate will be monitored with a HR monitor (Polar®, FT1, Polar Electro, Kempele, Finland). Using HR values at 4 min for the first recovery period, after squatting and after the first minute of recovery (for exact equation, see [64]), the Ruffier-Dickson Index will be calculated and used as a cardiorespiratory fitness index. Validity of this index has been reported by Sartor et al. [64].

#### Control variables

##### Job characteristics

Job characteristics will be assessed with 12 items from the French short version of the Copenhagen Psychosocial Questionnaire [86]. Designed to assess psychosocial risk factors at work, the original version has 46 items grouped in 24 scales referring to six dimensions: quantitative demands, autonomy, organization and leadership, horizontal relationships, work attitudes, and work-related well-being. In this study, we will remove scales that are inappropriate regarding the employment of future participants, and we will assess only one single-item per scale in order to reduce the length of this questionnaire. In addition, we will not measure the work-related well-being dimension which includes scales close to our primary and secondary outcomes. Thus, the “quantitative demands” dimension has three single-items assessing *workload* (“Do you have enough time for your work tasks?”), *work pace* (“Is it necessary to keep working at a high pace?”), and *cognitive demands* (“Does your work require that you remember a lot of things?”). The “organization and leadership” dimension has four single-items assessing *predictability* (“Do you receive all the information you need in order to do your work well?”), *recognition* (“Is your job recognized and appreciated by the management?”), *role clarity* (“Do you know exactly what is expected of you at work?”) and *social support from supervisor* (“Is your supervisor ready to listen to you about work problems”). The “horizontal relationships” dimension has one single-item assessing *social support from colleagues* (“Do your colleagues listen to your problems at work?”). The “autonomy” dimension has two single-items assessing *influence* (“Can you influence the amount of work assigned to you?”) and *possibilities for development* (“Do you have the possibility of learning new things through your work?”). The “work-individual interface” dimension has two single-items assessing *meaning of work* (“Do you feel that the work you do is important?”) and *job satisfaction* (“How pleased are you with your job as a whole, everything taken into consideration?”)*.* Participants will answer these items with a seven-point scale (1 = strongly disagree, 7 = strongly agree). The validity and reliability of this tool were satisfactory and have been reported by Dupret et al. [86].

##### Needs Satisfaction-Frustration at work

The satisfaction versus frustration of the three basic psychological needs at work will be measured using an adapted version of the Needs Satisfaction-Thwarting Scale [87]. It uses a seven-point bipolar response format, each boundary representing the frustration (− 3) versus the satisfaction (+ 3) of a need and the median value (0) corresponding to a neutral sentiment. Following the stem “In the past month, in my work I generally felt...”, participants will be asked to respond to 12 items assessing employee competence (4 items, e.g., “I felt competent (vs. incompetent) at my job”), autonomy (4 items, e.g., “I felt I was free to make my own decisions (vs. I had to follow the decisions that were made for me)”) and colleague relatedness (4 items, e.g., “I felt supported (vs. unsupported by my colleagues”) satisfaction and frustration. This scale demonstrated satisfactory factorial validity and reliability [87]. Using data from the baseline measurements, we will select the three most representative items according to their loading on each of the three factors to assess psychological recovery experiences (see below).

#### Manipulation Check

##### Instructor’s motivating style

To check that PA-NSS instructors display a motivating style that supports participants’ needs more than the PA-TS and the Theatre instructors, we will use measures from objective third-party observers (raters) scoring of the instructors’ motivating style and the participants’ self-reported perceptions of their instructor’s motivating style. To assess the *instructors’ observed motivating style*, all instructors will be recorded audio-visually in the eighth week. Two blinded researchers (i.e., raters) who are familiar with the SDT framework will be trained to score the instructors in terms of need-supportive and controlling motivational style used during the sessions. They will rate the instructors’ motivational style using a modified version of the Interpersonal Support in Physical Activity Consultations Observational Tool (ISPACOT; [88]). This tool assesses supervisors’ behaviors that capture four dimensions of motivational style: *autonomy support* (7 items; e.g., “The instructor encouraged the participants to put forward solutions to barriers”), *competence support* (4 items; e.g., “The instructor gave positive informational feedback to the participants for effort, improvement and task mastery”), *relatedness support* (2 items; e.g., “The instructor demonstrated dedication to and care for the participants”), and *interpersonal control* (8 items; e.g., “The instructor used controlling language with the participants” (e.g., “should, have to, must and ought to”). A seven-point scale (1 = *Not at all true*; 7 = *Very true*) will be employed to rate the degree to which the different behaviors are exhibited. Evidence for the reliability and convergent validity of the ISPACOT have been reported by Rouse [88]. To assess the *participants’ perception of the instructor’s motivating style* participants will be asked to evaluate their instructor’s motivating style during a session, using a slight adaptation of the ISPACOT (e.g., “The instructor listened carefully to how *I* wanted to do things” instead of “The instructor listened carefully to how *the participants* wanted to do things”).

##### PA intensity during sessions

To check that the two PA interventions produce more physical effort than the Theatre intervention, we will assess participants’ *objective* and their *perceived effort*. First, participants in the three intervention groups will be asked to wear a tri-axial accelerometer (ActiGraph LLC, Pensacola, FL, USA; ActiGraph GT3x) on the waist during a session in the sixth week of the intervention. The devices will be initialized with 30 s epochs and the data will be analyzed with Freedson’s algorithm [89]. Moderate to vigorous PA and energy expenditure will be calculated for each participant. Moreover, the *perceived effort* during the sessions will be assessed weekly with one single-item: “How intense was the effort you made during the last session?” (“Very slightly intense” vs. “Very intense”).

#### Weekly measures during the intervention period

During the ten weeks of the intervention period, at the end of the second session each week, all participants in the four conditions will be invited to complete a short questionnaire distributed in an individual booklet (for the PA-TS, PA-NSS, Theatre conditions) or an online (for the WLC condition) format. The questions will assess work-related variables (for all four groups) and intervention-related variables (only for the three intervention groups).

##### Work-related variables

Employees will be asked to complete a ten-item questionnaire after the second session each week, to assess their weekly *burnout at work* (i.e., three single-items from the SMBM; see above), *vigor at work* (i.e., three single-items from the SMVM; see above), *basic need satisfaction* versus *frustration at work* (i.e., three single-items from the Needs Satisfaction-Frustration at Work Scale; see above), and *workload* (one single-item “How heavy was your workload today?” used by Thøgersen-Ntoumanis et al., [28]). Scores will be rated using a bipolar visual analogue scale of ten centimeters with two opposing anchors (e.g., “never” vs. “always”; “very light” vs. “very heavy”).

##### Activity-related variables

Participants in the intervention groups will answer an additional eight-item questionnaire using the same bipolar visual analog scale as described above, in order to assess activity-related variables: *affect valence* (one item; “how do you feel after this session”, with the two opposing anchors “very bad” vs. “very good”) and *affect intensity* (one item; “how do you feel after this session”, with the two opposing anchors “low arousal” vs. “high arousal”), from Ekkekakis, Hall, Van Landuyt, and Petruzzello’s work [90]; five single-item measures assessing each of the *psychological recovery experiences* (see above); and the *perceived effort* (one item; “how intense was the effort you made during the last session?”, with the two opposing anchors “very slightly intense” vs. “very intense”).

### Statistical analysis

#### Analyses of Primary and secondary outcomes

Between-arm differences in changes on the primary and secondary outcomes will be analyzed using generalized linear mixed models (GLMM) according to intention-to-treat and per-protocol principles. Intention-to-treat analyses [91] refer to the effect of treatment “as assigned”, meaning that all participants who are randomized to a condition will be included in the analyses, regardless of dropout or missing values. By contrast, per-protocol analyses refer to the effect of treatment “as received”, excluding only participants who have less than 50% compliance to the 20 sessions in the intervention. GLMM has several advantages over repeated measures ANOVA as it considers the hierarchical nature of the data and can accommodate missing data [92]. To distinguish between short- and long-term intervention effects, two separate series of GLMM analyses were performed for each outcome variable. Two time points (pre and post) were used to determine short-term intervention effects and four time points (pre, post, three- and four-month follow-ups) were used to examine long-term intervention effects. Regarding the short-term intervention effects, differences in changes on the outcome variables as a function of the group allocation will be assessed with models including group, time, and group x time interaction as fixed effects. For the group allocation, contrasts analyses [93] will also be carried out to test our hypotheses (H1 – H4) specifically. For that, three contrasts will be computed. The first one will compare the WLC condition with the Theatre condition, PA-TS and PA-NSS conditions (respectively coded as − 0.75, + 0.25, + 0.25, + 0.25) to test whether doing a leisure activity (PA or Theatre conditions) at the workplace leads to a reduction in burnout and an improvement in vigor (H1a, H1b). The second contrast will compare the Theatre condition with the PA-TS and PA-NSS conditions (respectively coded as − 0.667, + 0.333, + 0.333, while WLC will be coded 0) to test whether PA is more effective than theatre condition (H2) in reducing burnout and improving vigor. Finally, the third contrast will compare the PA-TS condition with the PA-NSS condition (respectively coded as − 0.50, + 0.50, while WLC and Theatre conditions will be coded 0) to test whether the positive effect of PA is more pronounced when the instructor uses a need-supportive style (H3). The same analyses will be performed to examine if the effects of the intervention are still effective three months (T12) and six months (T13) after the end of the intervention period. Since there are more than two-time measurements per participant, we will allow random intercepts for participants and random linear slopes for the repeated measurements at the level of participants. These random effects will allow us to estimate each participant’s outcome variables and the rate of change of these outcomes over time. In all analyses we will control for a number of variables that could influence participants’ outcomes. These include job characteristics, and needs satisfaction-frustration at work. Finally, an estimate of the effect size for each outcome will be reported using the conditional pseudo R^2^, which will be computed using the MuMin package of the R software [94].

#### Analyses of Psychological and Physiological Mechanisms of the PA – Burnout/Vigor Relationship

Multiple mediation modelling will be used to examine the hypotheses that psychological (i.e., detachment, relaxation, mastery, control, relatedness, and positive affects experiences) and physiological (i.e., cardiorespiratory fitness) mechanisms partially or fully mediate the relationships between workplace PA or expressive activity on one hand and burnout and vigor on the other (H5). Two separate models will be generated with burnout and vigor as dependent variables. To handle the categorical independent variable (i.e., the experimental conditions), we will follow Hayes and Preacher’s [95] guidelines for calculating direct and indirect effects using a multicategorical predictor. Specifically, contrast coding will be used to examine the relative effect of workplace intervention (versus WLC), PA (versus Theatre condition) and PA with instructor using a need-supportive style (versus PA-TS) (see above). The scores of the mediating and dependent variables will be analyzed while controlling for the levels of measurement at baseline (i.e., the autoregressive effects). Autoregression allows the value of the variable at a previous time point to be statistical controlled, thereby reducing the likelihood of bias [96]. Bootstrapping procedures will be used to test the statistical significance of indirect effects of the proposed mediating variables [97]. Bootstrapping is a nonparametric resampling procedure for estimating indirect effects using adjusted (asymmetric) confidence intervals. This procedure is very useful in cases of multiple mediations, for which it is interesting to determine not only whether an indirect effect exists, but which mediator(s) contribute(s) significantly to the effect while controlling for other potential mediators. Point estimates and confidence intervals of relative indirect effects (total and specific) will be estimated from 5000 bootstrapped samples and 95% bias-corrected confidence intervals.

#### Analyses of Weekly trajectories

To investigate the trajectories of weekly variables over the intervention period (H6a, H6b, H6c), we will use growth models in a multilevel modelling framework. Two models will be tested for burnout and vigor respectively. They will contain a random intercept and a random linear slope for the occasion of measurement at the subject level. This random slope for occasion measurements will allow each participants’ growth trajectory over time to be estimated. A linear and quadratic effect of occasion measurements will be added as fixed effects to examine the linear and accelerated evolution of the outcomes over time. The three orthogonal contrasts will be added as fixed factors to the model, as well as their interaction terms with “time” and “time2”. Thus, we will examine if there is an effect of the intervention (H6a), the type of activity or need-supportive style (H6b) on the level of and rate of change in burnout and vigor over time. In all analyses we will control for variables that could influence participants’ outcomes, such as weekly needs satisfaction-frustration at work and weekly workload.

#### Analyses of Moderators

To examine whether participants’ experiences (i.e., detachment from work, relaxation, mastery, control, relatedness, or positive affects) during sessions moderate their well-being trajectories (H6c), a model comparable to the aforementioned models will be tested, except that the six experiences will be added as between-factors to the model and will also be modeled to interact with time (i.e., cross-level interaction). In these analyses, we will use each individual’s mean scores of the experiences during the 10 weeks of the intervention. Such a model, already used previously [66] will test if participants’ rate of change in well-being over time varies as a function of their average recovery experiences during the Nordic walking/ Theater sessions. To test if participants’ compliance to the intervention moderates their well-being trajectories (H7), we will also use a multilevel modelling framework. Two models will be tested for burnout and vigor respectively. This model is equivalent to the model testing weekly trajectories (see above), except that “compliance” will be added as a fixed factor in the model and will interact with other terms. This analysis strategy testing the moderation effect of compliance has been used in a study with a similar protocol design [66].

## Discussion

Implementing physical activity interventions in the workplace seems to be an effective way to prevent burnout and promote vigor among employees [9–11]. Therefore, this paper presents a four-arm randomized control trial protocol whose main objective is to evaluate beneficial effects of a PA intervention with a need-supportive style, on employees’ levels of burnout/vigor. More precisely, the study design will allow us to compare PA to another leisure activity (i.e., Theatre) and the influences of the context of PA practice (i.e., traditional vs. need-supportive style) on burnout/vigor. Furthermore, this study will also investigate several possible psychological and physiological mechanisms through which PA could influence burnout/vigor (i.e., mediators).

### Strengths and Limitations

This protocol presents three main strengths. The methodological robustness constitutes the main strength of this protocol. Indeed, the comparison of four groups (three experimental groups and a waiting list control group), the subject randomization, weekly measures, and objective measures of exercise intensity respond to recommendations made by some authors [33, 98], and add a significant contribution to the literature on the effectiveness of PA interventions to improve employee well-being. Moreover, to our knowledge, this study is the first to compare the effect of PA to the effect of another leisure activity (i.e., Theatre) on burnout and vigor. Previous interventions have either focused only on the effect of PA [37] or on the effect of mixed activity programs [23, 25–27] on indicators of well-being. Another strength of this protocol is that it focuses on mechanisms that mediate the effect of activity on burnout and vigor. More precisely, we will be able to examine if specific theory-based psychological constructs (i.e., detachment, relaxation, mastery, control, relatedness, and positive affects experiences) and a physiological component (i.e., cardiorespiratory fitness) are mediators of the relationship between conditions (i.e., PA-TS, PA-NSS, Theatre condition) and outcomes. In the past, only two interventions [28, 29] have addressed some of these mediators. Our study will then be the first to test the combined influence of these different mediators. Finally, another strength of this protocol is that it tests the additive effect of an instructor’s need-supportive style based on SDT on burnout and vigor. A previous study [28] has implemented an autonomy-supportive leadership style in a PA intervention in the workplace, and indicated positive effects on work-related affective states. However, the authors compared the changes in well-being of the intervention group to a control group, but not to a traditional style PA group. Consequently, their design prevents conclusions to be developed about the respective effect of the motivational style and PA. In the present study, we will compare a traditional style PA intervention to a need-supportive PA intervention in order to disentangle the specific effect of the motivation style and PA.

Despite the strengths and precautions taken in drafting this protocol, several limitations require caution. First, the study design is not “fully” blinded. While participants in the two PA groups will not be aware of the differences between these groups (i.e., PA-TS vs. PA-NSSS), and instructors for each PA condition will not be aware of the existence of another PA condition, participants and instructors in the Theatre and PA conditions will obviously not be blind to the activity being performed. Secondly, given that participants in the WLC group will be contacted, randomized, and will complete weekly questionnaires during the intervention period, they cannot be considered as a truly “untreated” group [99]. Indeed, answering questionnaires cause a behavioral change even if there is no intervention. As a result, we could anticipate that the effects found in this study will be underestimated compared to the true causal effect [66]. Third, we chose to only measure burnout and vigor once a week, during the second activity session to capture the dynamics of change. It might have been interesting to measures these variables both after the sessions and on days when there are no sessions, to compare days with activities to days without activities, in order to examine the effect of the activities on well-being on a daily basis. However, this procedure would have required an increase in the already large number of measures.

### Implication for practice

The study presented is significant and has direct applications in practice. If proven to be effective, it will make a unique contribution to the promotion of leisure activities and more specifically to the PA carried out in the workplace for the improvement of employee well-being. Indeed, this cost-effective, theory-grounded, scientifically validated intervention will benefit companies and employees. In addition, this intervention will contribute to scientific knowledge by highlighting the effectiveness of PA with an instructor trained to support needs, and the psychological and physiological mechanisms responsible for well-being improvement. Results of this study could be used by researchers to design new research protocols, and also by practitioners to implement more highly efficient programs in the workplace, and to improve the well-being of employees.

## Abbreviations

ISPACOT: Interpersonal support in physical activity consultations observational tool
PA: Physical activity
PA-NSS: Physical activity needs supportive style
PA-TS: Physical activity traditional style
SDT: Self-determination theory
SMBM: Shirom-Melamed burnout measure
SMVM: Shirom-Melamed vigor measure
WLC: Waiting list control
WOPAP: Workplace physical activity program
